# Relationships between core self-evaluation, leader empowering behavior, and job security among Jordan University Hospital nurses

**DOI:** 10.1371/journal.pone.0260064

**Published:** 2021-11-17

**Authors:** Ali M. Sulaiman, Othman A. Alfuqaha, Thana A. Shaath, Rawan I. Alkurdi, Rahmah B. Almomani

**Affiliations:** Department of Nursing, Jordan University Hospital, The University of Jordan, Amman, Jordan; University of Jyvaskyla, FINLAND

## Abstract

Nurses are facing real stressors due to patients’ needs and leaders’ demands. The aim of this study is to explore the perceived level of core self-evaluation (CSE), leader empowering behavior (LEB), and job security among Jordan University Hospital nurses in Amman, the capital of Jordan. Furthermore, it investigates the relationship between the selected variables. Differences of gender, educational level, experience, and site of work are also examined with job security. Moreover, it evaluates the contribution of CSE, LEB, gender, educational level, experience, and site of work in predicting job security among Jordan University Hospital nurses. A descriptive cross-sectional design was adopted for this study. A convenience sample of 214 nurses from Jordan University Hospital was completed the CSE scale, LEB scale, and job security scale. Descriptive statistics, Pearson correlation coefficient, t-test, one-way analysis of variance, and stepwise regression were used to analyze the results. The results indicate that job security is found to be at high level, whereas LEB and CSE are found to be at moderate levels among nurses. Significant positive relationships are found between CSE, LEB, and job security. Male nurses and medical/surgical floors reported higher levels of job security than female nurses and intensive care units. Finally, the results show that LEB and gender are significant predictors of job security among nurses. We suggest that managers of nurses should apply leadership behaviors in order to increase their job security and career empowerment.

## Introduction

Nurses are facing real stressors due to patients’ needs and leaders’ demands. These stressors refer to both external and internal conditions that are leading nurses to experience psychological burnout and job insecurity. External stressors that nurses are confronted by mainly relating to the healthcare environment, such as works hours, shortages of nurses, and conflicts with supervisors. Internal stressors relate to core self-evaluation (CSE); regardless of whether it is perceived negatively or positively, CSE remains a significant issue affecting nurses in the workplace [1].

CSE can be defined as a higher-order function used in self-assessment conclusions to assess many aspects of individuals such as personal worth, ability, competency, and emotional stability. It is widely recognized that CSE has four domains namely locus of control, emotional stability, generalized self-efficacy, and self-esteem [2, 3]. In previous studies, an increased level of CSE among nurses is associated with higher job satisfaction, lower job turnover, and improved job engagement and performance [4–6]. On the other hand, a decreased level of CSE among nurses is associated with higher job turnover, higher job burnout, and lower job accomplishments [7–9]. However, few studies have focused on CSE with leader empowering behavior (LEB).

LEB is a dynamic process that allows leaders or supervisors to delegate responsibility, authority, and autonomy toward their employees to enhance their self-control and self-management [10]. LEB mainly involves the cultivation of personal skills, the encouragement of innovation, and the sharing of information among staff nurses [11]. LEB among nurses reduces burnout levels and turnover intention [12, 13]. While job security among nurses leads to better engagement and emotional stability [14, 15].

The feeling of job security among nurses is mainly associated with their expectations and their predicted achievements in the future. When nurses suffer from job insecurity, this is cost-ineffective to both hospitals and patients [16, 17]. Job insecurity is associated with poor health, decreased personal well-being, and deteriorated work behavior [18, 19]. However, nurses’ demographic factors are poorly understood with job security; this suggests that researchers should investigate and better understand the association of demographic factors with job security.

Job insecurity is associated with core self-evaluation and leader empowering behavior [4, 20], but the relationships between CSE, LEB, and job security is poorly understood among nurses. Moreover, clarifying several demographic factors with job security among nurses is inadequately addressed. To date, there has been little attention given in such previous studies. To the best of our knowledge, none of these studies involved any research into the relationships between CSE, LEB, and job security among Jordan University Hospital nurses in Jordan, which is the aim of this study. Furthermore, the differences between gender, educational level, experience, and site of work are also examined with job security. Moreover, the contribution factors of CSE, LEB, and all studied demographic factors are investigated in this study to evaluate their association with job security and serve, as any of them can be important predictors of job security among Jordan University Hospital nurses. This study would typically contribute to nursing managers in determining the key factors of job security among their nurses.

## Methodology

### Study questions

1. What is the perceived level of core self-evaluation, leader empowering behavior, and job security among Jordan University Hospital nurses?
2. What are the relationships between core self-evaluation, leader empowering behavior, and job security among Jordan University Hospital nurses?
3. Are there statistically significant differences between several demographic factors including (gender, educational level, experience, and site of work) and job security among Jordan University Hospital nurses?
4. What are the contributions of core self-evaluation, leader empowering behavior, gender, educational level, experience, and site of work in predicting job security among Jordan University Hospital nurses?

### Study design

A descriptive cross-sectional study design was adopted to shed light on nurse’s perception of core self-evaluation, leader empowering behavior, and job security among nurses at Jordan University Hospital by determining the perceived levels of the selected variables and whether CSE, LEB, and nurses’ demographic factors influence job security.

### Study population and sampling procedure

The study population consisted of all nurses who worked at Jordan University Hospital, which is located in Amman, the capital of Jordan. Exclusion criteria included: nurses in outpatient clinics who almost had assistant degrees in nursing, nurses with less than 1 year of experience who were most likely rolling in training and educational sessions, and nurses who did not work with patients such as leaders and managers who were supposed to have job security and leader behaviors. Inclusion criteria of active participants in this study were nurses who provide direct care to the patients, agreed to voluntarily participate, and had at least one year of experience. To consider the entire nurses’ population in the selected hospital, 3 major areas conveniently were chosen (i.e., medical floors, surgical floors, and intensive care units) because it is readily accessible for researchers. These 3 major areas provide full different services and consist of various departments. For example, intensive care units represented pediatric, neonate, coronary, neuro, corona virus-19, surgical, and medical intensive care unit. In this regard, the total number of nurses in these major areas is almost 375. Due to the COVID-19 pandemic, we distributed the online self-reported questionnaire conveniently to each included department via a formal email. In order to detect the sample size, we included all nurses in these areas (375) that were deemed sufficient to attain the confidence interval of 95% and 5% margin of error [21]. Out of 375 nurses, a total of 228 participants returned the completed questionnaire with a response rate of 61%. A total of 14 incomplete online questionnaires were excluded and, hence, the analysis was performed on a final number of 214 questionnaires. After institutional review boards approved the study, the researchers went to each supervisor in the selected department to provide more details on the aim of this study, explain their right to refuse or participate, and to assure participant nurses that their responses would remain confidential and anonymous. The study was conducted from June to July 2020.

### Study tools

All published scales used in this study were translated and back-translated from the English language to the Arabic language, thus ensuring it would be appropriate for the participants. In this regard, permission was sought from the authors to use the selected scale. Moreover, the translated scales were presented to 4 arbitrators in nursing and psychology to provide their suggestions and views regarding the Jordanian context. In summary, the consent form, demographic factors, original published scales, and translated scales were included in the online survey.

#### Core self-evaluation

The CSE scale was used to measure the perceived level of CSE among nurses in Jordan. It is widely recognized that CSE has 4 domains: locus of control, emotional stability, generalized self-efficacy, and self-esteem. However, Judge et al. [2] developed a 12-item scale (3 items on each domain) to measure these domains collectively. The CSE scale has been shown to have good psychometric properties globally [22–24]. It is noteworthy to mention that 6 items in the CSE scale were rated in reverse order. Sampling adequacy was assessed using the Kaiser-Meyer-Olken (KMO) test and Bartlett’s test of sphericity. The KMO test was comprised at 0.73 [25]. The Bartlett’s test of sphericity was tested by Chi-square value (χ2) and it was χ2 = 559.66; df = 66; *P* < 0.001. These results were typically significant and suitable for factor analysis in this scale [26]. Correlation coefficients ranged from 0.58 to 0.91. The reliability was evaluated by applying the CSE scale (12 items) on a pilot study (40 nurses outside the study sample) in which Cronbach’s alpha was 0.71.

#### Leader empowering behavior

The LEB scale was used in this study to measure the perceived level of LEB among nurses in Jordan. The LEB scale consisted of 12 items adopted from [27]. Sampling adequacy was assessed by using the Kaiser-Meyer-Olken (KMO) test and Bartlett’s test of sphericity. The KMO test was comprised at 0.87 [25] and Bartlett’s test of sphericity was χ2 = 1295.39; df = 66; *P* < 0.001. These results were typically significant and suitable for factor analysis in this scale [26]. Correlation coefficients ranged from 0.57 to 0.91. The reliability was evaluated by applying the LEB scale on a pilot study (40 nurses outside the study sample) in which Cronbach’s alpha was 0.95.

#### Job security

The job security scale was used to measure the perceived level of job security among nurses. The job security scale developed by [28] and was utilized to assess their perception toward job security generally. The job security scale demonstrated good validity and reliability across 5 European countries [29]. However, the job security scale consisted of 9 items. Sampling adequacy was assessed by using the Kaiser-Meyer-Olken (KMO) test and Bartlett’s test of sphericity. The KMO test was comprised at 0.80 [25] and Bartlett’s test of sphericity was χ2 = 570.71; df = 36; *P* < 0.001. These results were typically significant and suitable for factor analysis in this scale [26]. Correlation coefficients ranged from 0.54 to 0.87. The reliability was evaluated by applying the job security scale on a pilot study (40 nurses outside the study sample) in which Cronbach’s alpha was 0.87.

All items of CSE, LEB, and job security were rated on a five-point Likert type scale from 1 "Strongly Disagree" to 5 "Strongly Agree". Negative items were rated in the reverse order. To assign the average score for the studied factors, mild average was used when rating ranges were from 1 to 2.33, moderate average was used when rating ranges were from 2.34 to 3.67, and high average was used when rating ranges were from 3.68 to 5.

### Ethical approval

The institutional review board committee in Jordan University Hospital approved this study (No:10/2020/9599).

### Statistical analysis

Statistical Package for the Social Sciences (SPSS V.23) was used to analyze the study results. Descriptive statistics were used to evaluate the perceived levels of CSE, LEB, and job security while Pearson correlation coefficient test was used to assess the relationships between CSE, LEB, and job security. Independent sample t-test and one-way analysis of variance were calculated to examine the differences of included demographic factors with job security. Finally, multiple linear regression analysis (i.e., stepwise method) was performed to assess the contribution of CSE, LEB, and several demographic factors including gender, educational level, experience, and site of work to identify whether any of these factors could serve as predictors of job security among Jordan University Hospital nurses. A *p*-value of 0.05 was deemed to be statistically significant.

## Results

### Participants

Table 1 presents the nurses’ demographic properties by calculating the descriptive statistics of frequencies and percentages.

**Table 1:** Properties of study participants (N = 214).

*Variables*	*Descriptive*	*Frequency*	*Percentage (%)*
Gender	Male	85	39.7
	Female	129	60.3
Educational level	Diploma degree	25	11.7
	Bachelor’s degree	154	72.0
	Postgraduate	35	16.4
Experience (years)	1 to 5	52	24.3
	5 to 12	113	52.8
	13 to 20	36	16.8
Site of work	more than 20	13	6.1
	Floor (medical/surgical)	99	46.3
	ICUs	115	53.7

ICUs: Intensive care units. M: Mean. SD: Standard deviation.

The participants in this study were, on average, predominantly female and nearly two-thirds were educated to the level of a bachelor’s degree. About fifty percent of nurses held 5 to 12 years of experience and worked in intensive care units. We presented the result section based on the study questions.

### Question one

What is the perceived level of core self-evaluation, leader empowering behavior, and job security among Jordan University Hospital nurses?

To answer this question, means, standard deviations, confidence interval levels, and overall levels are presented in Table 2.

**Table 2:** Means, standard deviations, confidence interval, and overall levels of the study variables.

*Variable*	*M*	*SD*	*95% Confidence interval for mean*		*Overall levels*
			*Lower bound*	*Upper bound*	
Leader empowering behavior	3.62	0.61	3.54	3.70	Moderate
Job security	3.83	0.46	3.77	3.90	High
Locus of control	2.89	0.61	2.81	2.97	Moderate
Emotional stability	3.39	0.72	3.29	3.49	Moderate
Generalized self-efficacy	3.43	0.39	3.38	3.48	Moderate
Self-esteem	3.85	0.51	3.78	3.92	High
The total level of Core self-evaluation	3.38	0.29	3.35	3.43	Moderate

M: Mean. SD: Standard deviation.

The overall mean score for CSE for nurses is (3.38 ± 0.29). Regarding the domains of CSE, self-esteem is found to be at high perceived level. Locus of control domain is found to be the lowest mean score (2.89 ± 0.61) with a moderate level. Other domains of CSE are found to be in moderate levels. Result shows that the perceived level of LEB among participated nurses is at moderate level. Job security exhibits the highest mean score with high perceived level compared to other factors studied.

### Question two

What are the relationships between core self-evaluation, leader empowering behavior, and job security among Jordan University Hospital nurses?

To answer this question, Pearson correlation coefficient test was performed and illustrated in Table 3.

**Table 3:** Pearson correlation coefficient between CSE, LEB, and job security among nurses (N = 214).

*Variable*	*CSE*	*LEB*
Job security	0.16	0.48
	0.02*	0.001**
Leader empowering behavior	0.14	
	0.04*	

LEB: Leader empowering behavior. CSE: Core self-evaluation.

*p-value ≤0.05.

**p-value <0.001.

Table 3 shows a positive relationship between job security and CSE (*r* = 0.16, *p* = 0.02). Similarly, job security is positively associated with LEB (*r* = 0.48, *p*<0.001). Perceived LEB has a significant relationship with CSE (*r* = 0.14, *p* = 0.04).

### Question three

Are there statistically significant differences between several demographic factors including (gender, educational level, experience, and site of work) and job security among Jordan University Hospital nurses?

Differences between selected demographic factors and job security are presented in Table 4.

**Table 4:** Differences between demographic factors and job security among Jordan University Hospital nurses (N = 214).

*Outcome*	*Variable*	*Descriptive*	*M*	*SD*	*r*	*95% Confidence interval*		*t/F-distribution*	*p-value*
						*Upper*	*Lower*		
Job security	Gender	Male	3.92	0.52	-0.16	4.03	3.81	2.30	0.02*
		Female	3.77	0.42		3.85	3.70		
	Educational level	Diploma degree	3.91	0.62		4.16	3.65		
		Bachelor’s degree	3.84	0.42	-0.09	3.91	3.77	0.85	0.43
		Postgraduate	3.75	0.51		3.93	3.58		
	Experience (years)	1 to 5	3.92	0.44		4.04	3.79		
		5 to 12	3.80	0.47		3.88	3.71		
		13 to 20	3.88	0.48	-0.09	4.04	3.72	1.63	0.18
		more than 20	3.65	0.40		3.89	3.41		
	Site of work	Floor (medical/surgical)	3.90	0.47		3.99	3.80	1.93	0.05*
					-0.13				
		ICU’s	3.77	0.45		3.86	3.69		

ICUs: Intensive care units. M: Mean. SD: Standard deviation. *r*: Pearson Correlation coefficient. t: t-test.

*p-value ≤0.05.

**p-value <0.001.

Table 4 reveals that gender is found to be associated with job security (*r* = -0.16, *p* = 0.02). Male nurses reported higher levels of job security than female nurses. Site of work is significantly associated with job security. Participant nurses in different floors such as medical and surgical are observed likelihood to engage in job security than nurses in ICUs. On the other hand, educational level and experience factors are not found to be significantly associated with job security among nurses.

### Question four

What are the contributions of core self-evaluation, leader empowering behavior, gender, educational level, experience, and site of work in predicting job security among Jordan University Hospital nurses?

To answer this question, multiple linear regression analysis (stepwise regression method) was performed to find the contribution of CSE, LEB, and several demographic factors including gender, educational level, experience, and site of work in predicting job security among nurses. The results are summarized in Table 5.

**Table 5:** Multiple linear regression analysis (stepwise regression) for job security predictors.

*Model*	*R*	*R Square*	*R Square change*	*Std*. *Error of the Estimate*	*Unstandardized Coefficients*		*t*	*Sig*.
					B	Std. Error		
1^a^	0.48	0.23	0.23	0.408	2.518	0.168	14.96	<0.001**
					.363	0.046	7.92	<0.001**
2^b^	0.50	0.25	0.02	0.404	2.738	0.193	14.16	<0.001**
					.359	0.045	7.89	<0.001**
					-.127	0.057	-2.25	0.025*

Dependent Variable: Job security. Sig: Significant at

*p-value ≤0.05.

**p-value <0.001.

^a^ Predictors: Leader empowering behaviors.

^b^ Predictors: Leader empowering behaviors, Gender.

Table 5 presented the predictors of job security. The results show that the two factors significantly predicting job security which are LEB and gender, with total variation of 25.0%. LEB is found to account for 23.0% of the variance in job security (R^***2***^ = 0.23, *p <*0.05). Gender is also found to be a significant predictor of job security, accounting for 2% of the variance in job security (R^***2***^ = 0.02, *p <*0.05). On the other hand, the model of stepwise regression has indicated that CSE, educational level, experience, and site of work is not considered to be significant predictors of job security among nurses (Table 6).

**Table 6:** Results of the excluded variables according to the multiple linear regression analysis for job security.

*Model*	*Variables*	*Beta In*	*t*	*Sig*.	*Partial Correlation*
**1**	Core self-evaluation	0.100^b^	1.648	0.101	0.113
	Gender	-0.135-^b^	-2.250-	0.025	-0.153-
	Educational level	-0.084-^b^	-1.394-	0.165	-0.096-
	Experience	-0.099-^b^	-1.648-	0.101	-0.113-
	Site of work	-0.022-^b^	-.370-	0.712	-0.025-
**2**	Core self-evaluation	0.108^c^	1.801	0.073	0.123
	Educational level	-0.084-^c^	-1.410-	0.160	-0.097-
	Experience	-0.087-^c^	-1.454-	0.147	-0.100-
	Site of work	-0.022-^c^	-.366-	0.715	-0.025-

^b^ Predictors in model 1: Leader empowering behavior.

^c^ Predictors in model 2: Leader empowering behavior, Gender.

## Discussion

We found that Jordan University Hospital nurses perceive a high level of job security. The perceived levels of LEB and CSE are found to be at moderate level. Job security is positively correlated with LEB and CSE. Site of work is deemed necessary for job security. Perceived LEB and gender emerged as predictors of job security.

Potential explanations of moderate level of CSE may occur due to nurse beliefs and values about their abilities to have control over the situation that happens in the workplace. There is evidence that Jordan University Hospital nurses have appeared personality trait of external locus of control than internal locus of control which explains that nurses depend on outer forces and abilities of other [30]. High perceived levels of CSE among nurses are mainly associated with better decision making, achieving goals, and improving performance in the workplace [31]. In contrast, low perceived levels of CSE are associated with lower satisfaction levels, lower control, and limited abilities [32]. Analyzing the core self-evaluation domains, self-esteem domain is only found to be at high levels among participated nurses. This is indicative of improving self-control, performing effective health care, and successfully facing nursing tasks [31] which could lead to good self-esteem. This result is in line with the study of Alfuqaha et al. [7], which also reported a moderate level of self-evaluation among nurses in Jordan.

It could not be surmised that LEB is at moderate level since nurses in Jordan are under constraints in the form of burnout, time pressure, lack of achievement, and imbalance between job demands and job resources [12, 33]. LEB has a direct effect on enhancing work effectiveness and eliminating workloads [34]. A positive leadership behavior among executive directors/managers of nurses ensures work engagement for their employees [35], fosters trust, and improves the quality of care [36]. On the other hand, with a lack of leader support recognized as the main cause of role conflict with colleagues, role ambiguity, and job dissatisfaction [37, 38].

Surprisingly, our study indicated that job security among participated nurses in this study is found to be at high levels. Potential explanations for higher levels of job security refer to stability and higher income salaries compared to other hospitals in Jordan. Another explanation may be related to the permanent contract system (automatically renewed) at Jordan University Hospital compared to other hospitals (mainly hospitals in private sectors) which depend on their performance and their productivity. This finding is in agreement with the study of [39] and disagreement with the study of [40] which reported a moderate level of job security among nurses.

Job security has been positively associated with nurses’ perception of CSE and LEB. This could be said that higher job security makes nurses feel more empowered, with a greater CSE. This finding suggests that job security among nurses has a direct effect not only on their self-evaluation but also on their empowerment and professional status. Meanwhile, a sense of control, positive leadership, and support by nursing supervisors tend nurses to be less job insecurity and intention to leave the profession [41, 42]. Previous studies showed that work environment [40], health care systems [43], and work conditions [16] were reported barriers of job security among nurses. Another study in CSE was found to be negatively associated with job security [44].

Furthermore, we found that LEB is associated with CSE. Similar to this finding, previous studies have found that LEB is positively associated with organizational commitment [45], job engagement [46], and job crafting [47].

We found that the male gender of a nurse the more he is to job security than the female gender. This could be related to the gender roles of male nurses to secure the basic elements of life. Moreover, floor nurses (medical/surgical) are more likely to experience job security than ICU nurses. This finding is not in parallel with the study of [48] which showed that gender was not a significant predictor of job insecurity.

LEB is key to achieving and ensuring job security among nurses since LEB is the main predictor of job security. Our findings as well as previous studies reflect in fact that LEB has encouraged nurses to enhance job satisfaction [49], make the right decisions [31], innovate their performance [50] and reduce burnout levels [51]. Gender of nurses also renders a mere contribution to job security. Regarding other selected demographic factors, none of them were significantly predicted by job security among participated nurses in this study which needs further explanations by qualitative studies in the future. To the extent that any study has limitations. Accordingly, our study recruited one hospital, used a convenience procedure, applied online self-reporting measurement to collect the data, and did not select several demographic factors such as age and geographical area which limits the generalization of current findings.

## Conclusions and recommendation

Nurses in the selected hospital have higher job security and moderate CSE and LEB. Significant positive relationships are found between CSE, LEB, and job security. The job security of nurses is associated to their male and their site of work. LEB and gender are significant predictors of job security. The effect of LEB on job security proves to be contributed towards enhancing work conditions and preventing experience of insecurity among nurses. On the other hand, enhancing the role of personality factors such as self-esteem and self-efficacy can be seen to have an effect on job security among nurses. We suggest that managers of nurses should apply leadership behaviors in order to increase their job security and career empowerment. Moreover, we recommend further research be conducted into CSE among healthcare providers in Jordan. Finally, future studies evaluating LEB, job security, and CSE are needed for different professions in Jordan.
